# Effects of Exogenous Organic Acids on Cd Tolerance Mechanism of *Salix variegata* Franch. Under Cd Stress

**DOI:** 10.3389/fpls.2020.594352

**Published:** 2020-10-23

**Authors:** Songlin Zhang, Hongchun Chen, Danni He, Xinrui He, Ya Yan, Kejun Wu, Hong Wei

**Affiliations:** ^1^Key Laboratory of Eco-environments in Three Gorges Reservoir Region (Ministry of Education), Chongqing Key Laboratory of Plant Ecology and Resources Research in Three Gorges Reservoir Region, School of Life Sciences, Southwest University, Chongqing, China; ^2^Key Laboratory of Reservoir Aquatic Environment, Chongqing Institute of Green and Intelligent Technology, Chinese Academy of Sciences, Chongqing, China; ^3^Guizhou Provincial Water Conservancy Research Institute, Guiyang, China

**Keywords:** cadmium, *Salix variegata*, organic acid, antioxidant enzymatic system, non-protein sulfhydryl compounds, gene expression

## Abstract

Chelate induction of organic acids has been recognized to enhance metal uptake and translocation in plants, but the underlying mechanism remains unclear. In this study, seedlings of *Salix variegata* were hydroponically exposed to the combinations of Cd (0 and 50 μM) and three exogenous organic acids (100 μM of citric, tartaric, or malic acid). Plant biomass, antioxidant enzymes, non-protein thiol compounds (NPT) content, and the expression of candidate genes associated with Cd accumulation and tolerance were determined. Results showed that Cd significantly inhibited plant biomass but stimulated the activity of antioxidant enzymes in the roots and leaves, while the lipid peroxidation increased as well. Respective addition of three organic acids greatly enhanced plant resistance to oxidative stress and reduced the lipid peroxidation induced by Cd, with the effect of malic acid showing greatest. The addition of organic acids also significantly increased the content of glutathione in the root, further improving the antioxidant capacity and potential of phytochelatin biosynthesis. Moreover, Cd induced the expression level of candidate genes in roots of *S. variegata*. The addition of three organic acids not only promoted the expression of candidate genes but also drastically increased Cd accumulation in *S. variegata*. In summary, application of citric, tartaric, or malic acid alleviated Cd-imposed toxicity through the boost of enzymatic and non-enzymatic antioxidants and candidate gene expression, while their effects on Cd tolerance and accumulation of *S. variegata* differed.

## Introduction

In recent decades, anthropogenic activities such as industrialization, mining, fossil fuel combustion, and sewage treatment have caused a large increase in the content of metals (e.g., lead, zinc, copper, manganese, and cadmium) in the soil and resulted in serious environmental problems (Wong, 2003). Among all heavy metals, cadmium (Cd) is a highly toxic, biologically undesirable carcinogen, ranking seventh among the top 20 toxic elements (Sanita di Toppi and Gabbrielli, 1999; Gill and Tuteja, 2011). Cd can be easily absorbed and accumulated by plants because of its high mobility and water solubility (Liu J. et al., 2013; Liu X. et al., 2013). It impairs a series of important physiobiochemical processes and severely inhibits the growth and development of plants (Gill and Tuteja, 2010; Dias et al., 2013). As a non-redox metal, Cd cannot participate in the Fenton-type reaction, but it can cause oxidative stress by the lipid peroxidation and overproduction of reactive oxygen species (ROS) (Wang et al., 2004; Heyno et al., 2008). ROS are extremely harmful to membrane lipids and cellular macromolecules. They oxidize the lipid and membrane and inactivate several enzymes, leading to the eventual cell death (Cuypers et al., 2010). Cd in the environment would also disrupt the balance of ecosystem through accumulation and transmission in the food chain, posing a tremendously negative impact on the ecological environment and human health.

In some Cd-tolerant plant species and hyperaccumulators, a succession of physiochemical mechanisms related to Cd resistance and detoxification have gradually been formed. For example, the effective ROS defense system, chelation of metals by ligands, cellular compartmentalization, and genetic regulation of metal transporters (Memon and Schroder, 2009; Yuan et al., 2012) can all be employed to alleviate Cd toxicity, as well as other heavy metal ions. In plant cells, enzymatic antioxidants mainly composed of superoxide dismutase (SOD), catalase (CAT), and ascorbate peroxidase (APX) and non-enzymatic antioxidants composed of phenols, carotenoids, and glutathione (GSH) coordinate jointly to scavenge ROS and minimize oxidative stress (Gill and Tuteja, 2010; Mahmud et al., 2018). In addition, studies on rice, wheat, and *Arabidopsis* have found that thiol compounds such as GSH, phytochelatin (PC), and cysteine (Cys) can sequester free Cd into plant cell vacuoles to inactivate Cd (Hartley-Whitaker et al., 2001; Gallego et al., 2012). Four main types of proteins, namely, ZRT-IRT-like protein, natural resistance-associated macrophage protein (NRAMP), heavy metal ATPase (P1B-ATPase or CPx-type ATPase), and metal tolerance protein (MTP), play key roles in the sequestration and detoxification of heavy metals (Hanikenne et al., 2008; Pottier et al., 2015). Metallothioneins (MTs) and PCs also synergistically mediate the toxicity of Cu and Cd in plants (Guo et al., 2008). For example, Lee et al. (2004) confirmed that the MT *AtMT2a* and *AtMT3* were responsible for Cd resistance in the intact cell of *Arabidopsis*.

In recent years, “chelate-induced phytoremediation” has attracted widespread attention in the field of phytoremediation (Han et al., 2016). Compared with man-made synthetic chelating agents, natural low-molecular-weight organic acids, a critical group of plant secondary metabolites, such as citric acid, tartaric acid, and malic acid, have higher degradability and lower toxicity and are more in favor of the requirement of environmental friendliness (do Nascimento et al., 2006). Organic acids can increase the solubility and bioavailability of heavy metals in the medium through promoting the desorption of metal ions and forming soluble complexes with metal ions, thus enhancing metal absorption by plants (Wuana et al., 2010; Chen et al., 2020). Moreover, studies have shown that organic acids also play an important role in tricarboxylic acid cycle metabolism, photosynthetic and respiration, nutrient uptake, and toxic metal detoxification in plants (Schulze et al., 2002; Araujo et al., 2012). Under Cd stress, the addition of malic acid can alleviate Cd-induced oxidative damage by regulating the enzymatic and non-enzymatic antioxidants in *Miscanthus saccharifloru*s (Guo et al., 2017).

*Salix variegata* Franch. is a perennial shrub of Salicaceae family. It has been proven to have broad application prospects in phytoremediation engineering due to its fast growth rate, strong reproductive capacity, and great Cd tolerance and enrichment ability in Cd contaminated soil (Jia et al., 2011, 2012; Sun et al., 2012). Previous studies have found that respective application of citric, tartaric, and malic acid significantly increased the proportion of free Cd^2+^, a favorable form for plants to absorb, in the nutrient solution, and Cd toxicity was substantially mitigated with respect to plant biomass and photosynthesis of *S. variegata* (Chen et al., 2019, 2020). However, the underlying mechanism of how organic acids impact Cd tolerance of *S. variegata* at the physiological and genetic levels is still unclear. Therefore, this study aimed to investigate the effects of individual addition of three organic acids, namely, citric acid, tartaric acid and malic acid, on the physiochemical responses and corresponding gene expression related to Cd accumulation in *S. variegata* under Cd stress. Our study would provide the scientific basis for promoting the application of chelate-induced phytoremediation technology.

## Materials and Methods

### Plant Material and Culture Conditions

The seedlings of *S. variegata* were collected from the bank of Jialing River in Chongqing (29°41′2′′ N, 106°26′56′′ E) on March 30, 2018. Before the experimental treatments, seedlings were transplanted and cultivated with conventional field management, including regular watering and weeding, in the ecological garden of Southwest University. Seedlings with uniform growth parameters were then transplanted into the polyethylene pots (ϕ18 cm × h 16 cm) and transferred to a light chamber on May 11, 2018. Each pot was added with 1.7 L of 1/2 modified Hoagland’s nutrient solution (pH 5.5) in which the detailed recipe was based on Nawaz et al. (2017). The day and night air temperatures were maintained at 25 and 20°C, respectively. The day length was 16 h, and the light intensity was 95 μmol ⋅ m^–2^ ⋅ s^–1^.

### Treatment Test

After 1 week of preculture, the seedlings were randomly divided into five treatments: (1) CK group, no Cd or organic acid addition; (2) Cd group, 50 μM of Cd; (3) Cd + citric acid group, 50 μM of Cd and 100 μM of citric acid; (4) Cd + tartaric acid group, 50 μM of Cd and 100 μM of tartaric acid; and (5) Cd+ malic acid group, 50 μM of Cd and 100 μM of malic acid. The concentrations of Cd and organic acids were chosen based on the typical content in the natural environment and the results of previous studies (Chen et al., 2020; Guo et al., 2017). All treatments contained three replicates. Cd was added in an aqueous solution as CdCl_2_ ⋅ 2.5 H_2_O, and all organic acids were added as aqueous solutions of pure acid. The pH value of nutrient solution in each treatment was 4.09–6.00, and free Cd^2+^ was detected as the primary form, accounting for 89.6–96.6% of the total Cd (Chen et al., 2019, 2020). During the experimentation period, the nutrient solution was continuously aerated and renewed weekly.

### Assay of Plant Biomass and Cd Accumulation

Plants were harvested at 4 weeks after treatment. The roots were first immersed in 20 mM EDTA-Na_2_ solution for 5 min, and then the seedlings were rinsed with distilled water and divided into roots, stems, and leaves. Each part was dried at 80°C to constant weight and gravimetrically measured. Dry sample 0.05 g was digested with concentrated mixed acids (HNO_3_:H_2_O_2_ = 3:1), and the concentration of Cd was determined by an inductively coupled plasma-optical emission spectrometry (Thermo Fisher, United States).

### Assay of Malondialdehyde and Antioxidant Enzymes

After 4 weeks of treatment, malondialdehyde (MDA) concentration in the roots and leaves of *S. variegata* was measured according to Guo et al. (2004). Briefly, 0.2 g of fresh tissues was homogenized in 10 mL of 10% (vol/vol) trichloroacetic acid (TCA) containing 0.25% (wt/vol) 2-thiobarbituric acid (TBA). The homogenate was heated at 95°C for 30 min and then quickly cooled on ice. After centrifugation at 10,000 × *g* for 10 min, the absorbance of the supernatant was measured at 450, 532, and 600 nm. The value for nonspecific absorbance at 600 nm was subtracted. MDA concentration was expressed as mmol ⋅ g^–1^ FW by an extinction coefficient of 155 mM^–1^ ⋅ cm^–1^.

Antioxidant enzymes including SOD, peroxidase (POD), CAT, and APX in roots and leaves of *S. variegata* were determined. Fresh sample 0.5 g was frozen in liquid nitrogen and then homogenized using 5 mL of 50 mM potassium phosphate buffer (pH 7.0, containing 0.1 mM EDTA) with prechilled mortar and pestle. The homogenate was centrifuged at 15,000 × *g* for 20 min at 4°C, and the supernatant was used for the assay of enzyme activities. SOD activity was determined according to Beauchamp and Fridovich (1971). The 3 mL of reaction mixture contained 50 mM potassium phosphate buffer (pH 7.0), 13 mM methionine, 75 μM nitroblue tetrazolium (NBT), 2 μM riboflavin, and 0.1 mL of enzyme extract. One unit of SOD activity was estimated as 50% reduction of NBT at 560 nm. POD activity was determined using guaiacol as a substrate (Nickel and Cunningham, 1969). The 3 mL of reaction mixture consisted of 25 mM phosphate buffer (pH 7.0), 0.05% guaiacol, 1.0 mM H_2_O_2_, and 0.1 mL of enzyme extract. The increase in guaiacol oxidation was recorded within 3 min at 470 nm. CAT activity was determined spectrophotometrically by the method of Aebi (1984) with some modifications. The 3 mL of reaction mixture contained 50 mM potassium phosphate buffer (pH 7.0), 10 mM H_2_O_2_, 0.1 mM EDTA, and 0.1 mL of enzyme extract. The CAT activity was assayed by monitoring the absorbance decrease at 240 nm as a consequence of H_2_O_2_ disappearance. APX activity was determined according to Nakano and Asada (1981). The 3 mL of reaction mixture included 25 mM potassium phosphate buffer (pH 7.0), 10 mM H_2_O_2_, 0.1 mM EDTA, 0.25 mM ascorbate, and 0.1 mL of enzyme extract. The oxidation activity of ascorbate was observed by the variation in absorbance at 290 nm. The MDA concentration was determined by the TCA-TBA method (Guo et al., 2004).

### Analysis of NPTs

The total NPT was measured according to Duan et al. (2011). One gram of fresh tissue was ground with liquid nitrogen and homogenized with 3 mL 5% (wt/vol) sulfosalicylic acid containing 6.3 mM diethylenetriaminepentaacetic acid. The homogenate was then centrifuged at 12,000 × *g* for 15 min at 4°C, and the supernatant was collected for determination of thiols. For NPT analysis, 0.2 mL of supernatant was mixed with 2.65 mL of 0.25 mM Tris–HCl and 0.15 mL of 10 mM 5,5-dithio-2-nitrobenzoic acid (DTNB). The absorbance was measured after 3 min in a spectrophotometer (UV-2550, SHIMADZU, Japan) at 412 nm (30°C). A reagent blank was referred to correct the absorbance of DTNB. NPT concentration was calculated using an extinction coefficient of 13,600 M^–1^ ⋅ cm^–1^. For GSH determination, 1 g of frozen tissue was homogenized in 5% TCA (vol/vol) solution and then centrifuged at 12,000 × *g* for 15 min at 4°C to obtain the supernatant. Determination of GSH was performed spectrophotometrically by the method of Wang et al. (2016), and the absorbance at 412 nm was recorded. The concentration of non-GSH NPT was calculated as follows: non-GSH NPT = NPT − GSH (Duan et al., 2011).

### Quantitative Real-Time Polymerase Chain Reaction Analysis

The *S. variegata* transcriptome database (unpublished data) was mined for genes involved in Cd transportation and detoxification pathways. The gene expression profiles include *NRAMP5* of the NRAMP family, metal tolerance protein *MTP1* and *MTP4*, P-type metal ATPase *HMA1*, *HMA3*, and *HMA5*, MT protein *MT1A* and *MT2B*, and GSH γ-glutamylcysteinyl transferase1 *PCS1*.

#### RNA Extraction

Total RNA was extracted using mirVana^TM^ miRNA ISOlation Kit (Ambion, Carlsbad, CA, United States) according to the manufacturer’s specifications. The quality and yield of RNA were determined using a NanoDrop 2000 spectrophotometer (Thermo Scientific, United States) and evaluated using agarose gel electrophoresis stained with ethidium bromide.

#### Real-Time Quantitative Reverse Transcription–Polymerase Chain Reaction

Quantification was performed with a two-step reaction process: reverse transcription (RT) and polymerase chain reaction (PCR). Briefly, 8 μL mixture containing 0.5 μg RNA, 2 μL of 4 × *g* DNA wiper mix, and nuclease-free H_2_O was reacted in a GeneAmp^®^ PCR System 9700 (Applied Biosystems, United States) for 2 min at 42°C. The mixture was then added with 2 μL of 5 × HiScript II Q RT SuperMix II^a^ and further reacted for 10 min at 25°C, 30 min at 50°C, and 5 min at 85°C. The 10 μL RT reaction mix was diluted 10 times in nuclease-free water and restored at −20°C. Real-time PCR was performed using LightCycler^®^ 480II Real-time PCR Instrument (Roche, Swiss) with a 10 μL PCR reaction mixture consisting of 1 μL of cDNA, 5 μL of 2 × QuantiFast^®^ SYBR^®^ Green PCR Master Mix (Qiagen, Germany), 0.2 μL of forward primer, 0.2 μL of reverse primer, and 3.6 μL of nuclease-free water. Reactions were incubated in a 384-well optical plate (Roche, Swiss) at 95°C for 10 min, followed by 40 cycles of 95°C for 10 s and 60°C for 30 s. Each sample was run in triplicate for analysis, and three replicates in each treatment were determined. At the end of the PCR cycles, melting curve analysis was performed to validate the specific generation of the expected PCR product. The primer sequences were designed in the laboratory and synthesized by Generay Biotech (Generay, PRC) based on the mRNA sequences obtained from the NCBI database (Table 1).

**TABLE 1:** Gene primers for RT-qPCR.

Gene name	Forward primer	Reverse primer
NRAMP5	TCATTTGAGCTTCCATTCGC	GGTCCCATCTTGGTGTTACT
MTP1	CAAGTTCTCCAGCAAATACG	CACGCCAACAGACTGAATTA
MTP4	GTAGAAGCTCGGGAAACAG	GCAACCAATCCTACCACAT
HMA1	TTGTGCAGGATGATATTAACCG	TTGGATAGGTGGCTGTGT
HMA3	TCCTCAGAAAGCAGTCATAGC	ATCGACAACAACTCCATCAAT
HMA5	ACGTGTTGATCGCTTTAGGAA	ATCCGTGGACTCAAATATTGG
MT1A	AGGTGTTGCACCAGTTAAG	CCATTCTCAGCACCGAAG
MT2B	CCTGAGTTTCTCCGAGACC	CATCCTAACCGGAGCGACA
PCs1	GAAGTGATATGGTCAATGGTCG	CAATGTGGTATGTTTCCGGT
GAPDH–*Salix matsudana*	TAGGCTGTTGGAAAGGTTC TGCCA	TGCCTTCTTCTCAAGCCT GACAGT

*The expression levels of mRNA were normalized to *Salix matsudana*–GAPDH and were calculated using the 2^–ΔΔ*Ct*^ method (Livak and Schmittgen, 2001).*

### Statistical Analyses

One-way analysis of variance and Tukey test were performed for plant biochemical data using SPSS 22.0 software, and 5% probability level was applied to distinguish significant difference among treatments. All results were expressed as the mean ± SE from three replicates.

## Results

### Plant Biomass and Cd Accumulation

As shown in Table 2, the tissue biomass of *S. variegata* was significantly inhibited by Cd except the leaf, compared with CK. Individual addition of the three exogenous organic acids markedly increased plant biomass as well as root and stem biomass under Cd stress. Among them, the root, stem, and total plant biomass in the Cd+ malic acid treatment were 201, 241, and 208% of that in the Cd treatment group, respectively, exhibiting the greatest effect on the mitigation of Cd-induced biomass of *S. variegata* (Table 2).

**TABLE 2:** Effect of organic acids on the biomass of *S. variegata* under Cd stress.

Treatments	Plant biomass (g)			
	
	Root	Stem	Leaf	Total
CK	1.30 ± 0.06^b^	1.15 ± 0.03^ab^	0.68 ± 0.06^a^	3.12 ± 0.1^b^
Cd	0.83 ± 0.02^a^	0.75 ± 0.07^a^	0.53 ± 0.02^a^	2.12 ± 0.15^a^
Cd + citric acid	1.34 ± 0.11^b^	1.17 ± 0.07^ab^	0.71 ± 0.05^a^	3.22 ± 0.25^b^
Cd + tartaric acid	1.29 ± 0.15^b^	1.47 ± 0.05^bc^	0.95 ± 0.04^a^	3.71 ± 0.26^bc^
Cd + malic acid	1.67 ± 0.10^b^	1.82 ± 0.04^c^	0.92 ± 0.07^a^	4.41 ± 0.17^c^

*Values are mean ± SE (*n* = 3). Values with different letters indicate significant differences between treatments at *P* < 0.05.*

Cd concentration in tissues of *S. variegata* varied with the types of organic acids added (Table 3). As compared with the Cd group, treatment with citric acid significantly increased the root Cd by 37%, whereas the stem Cd was notably reduced to 75% of that in the Cd-treated group by malic acid. In the leaves, the addition of tartaric acid greatly increased Cd concentration by 81% relative to the Cd treatment group. Moreover, respective addition of organic acids significantly increased Cd accumulation in tissues of *S. variegata*, regardless of the types. The total plant Cd amounts in citric, tartaric, and malic acid treatments were 209, 190, and 178% of that in Cd treatment group, respectively (Table 3).

**TABLE 3:** Effect of organic acids on Cd concentration and accumulation of *S. variegata* under Cd stress.

Treatments	Cd concentration (mg ⋅ kg^–1^)			Cd accumulation (μg ⋅ plant^–1^)			
		
	Root	Stem	Leaf	Root	Stem	Leaf	Total
Cd	232.43 ± 13.10^ab^	33.77 ± 1.53^bc^	4.03 ± 0.18^ab^	191.85 ± 6.38^a^	25.76 ± 4.85^a^	2.15 ± 0.04^a^	219.76 ± 4.21^a^
Cd + citric acid	320.23 ± 12.44^c^	27.77 ± 1.02^ab^	3.10 ± 0.17^a^	425.85 ± 24.69^b^	32.43 ± 5.85^ab^	2.19 ± 0.33^a^	460.47 ± 18.53^b^
Cd + tartaric acid	274.97 ± 7.78b^c^	39.80 ± 1.86^c^	7.33 ± 0.32^c^	351.06 ± 31.52^b^	58.70 ± 4.25^c^	6.84 ± 1.33^b^	416.60 ± 34.05^b^
Cd + malic acid	204.87 ± 8.37^a^	25.23 ± 0.52^a^	5.47 ± 0.37^b^	340.85 ± 18.43^b^	45.78 ± 0.36^bc^	5.02 ± 0.63^ab^	391.65 ± 19.32^b^

*Values are mean ± SE (*n* = 3). Values with different letters indicate significant differences between treatments at *P* < 0.05.*

### Lipid Peroxidation and Antioxidant Enzymes

**FIGURE 1:**
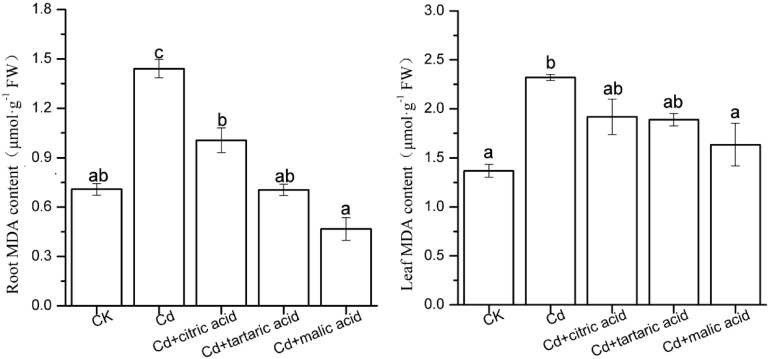
Effects of 100 μM of exogenous organic acid (citric, tartaric, or malic acid) on lipid peroxidation of the roots and leaves of *S. variegata* exposed to 50 μM of Cd for 4 weeks. Values are means ± SE (*n* = 3). Different letters indicate significant difference between treatments at *P* < 0.05.

The MDA content of membrane lipid peroxidation in roots and leaves is shown in Figure 1. Under Cd stress, the MDA content increased by 103 and 69% in roots and leaves, respectively, as compared with CK. Respective addition of three organic acids alleviated lipid peroxidation in plant roots and leaves at different degrees. Specifically, the MDA content in roots of *S. variegata* was significantly decreased in the treatment group of citric, tartaric, and malic acid by 30, 51 and 68%, respectively, relative to Cd treatment group. While the MDA content in leaves only decreased to 71% in the treatment of malic acid as compared with Cd treatment group (Figure 1).

**FIGURE 2:**
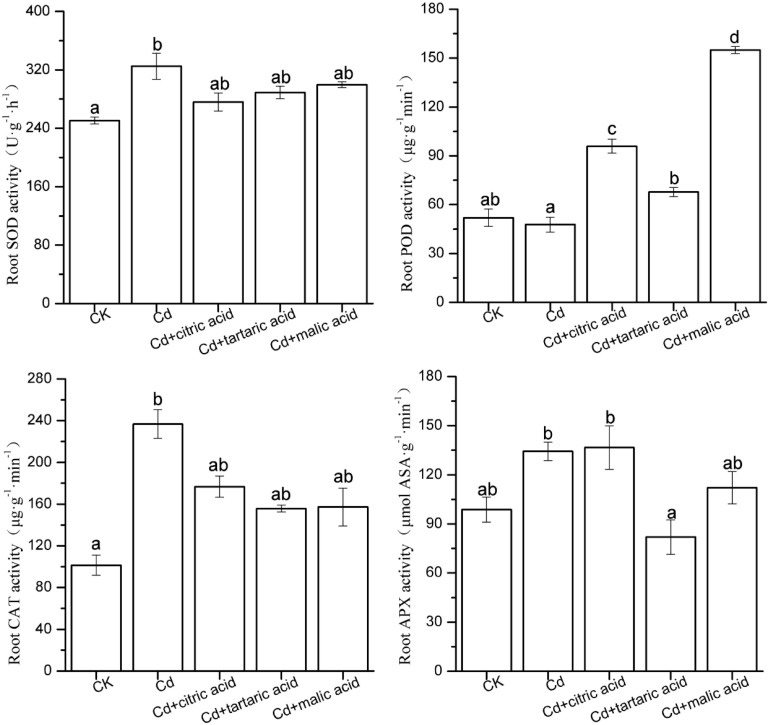
Effects of 100 μM of exogenous organic acid (citric, tartaric, or malic acid) on the antioxidant enzymes in roots of *S. variegata* exposed to 50 μM of Cd for 4 weeks. Values are means ± SE (*n* = 3). Different letters indicate significant difference between treatments at *P* < 0.05.

**FIGURE 3:**
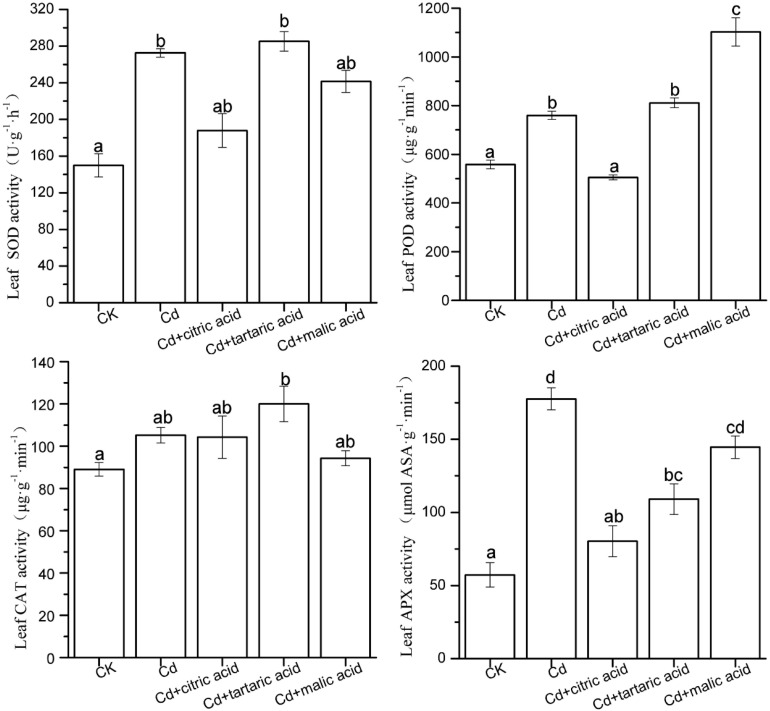
Effects of 100 μM of exogenous organic acid (citric, tartaric, or malic acid) on the antioxidant enzymes in leaves of *S. variegata* exposed to 50 μM of Cd for 4 weeks. Values are means ± SE (*n* = 3). Different letters indicate significant difference between treatments at *P* < 0.05.

To confirm whether the effect of organic acids on MDA content in the roots and leaves of *S. variegata* under Cd stress was due to the activation of antioxidant systems, we measured the activities of key antioxidant enzymes (SOD, POD, CAT, and APX; Figures 2, 3). In roots, the activities of SOD and CAT significantly increased by 30 and 133%, respectively, under Cd stress. Individual addition of citric acid and malic acid greatly increased the POD activity to 201% and to 324% of that in Cd treatment group, respectively. Tartaric acid treatment significantly increased the POD activity by 42%, whereas the APX activity was substantially reduced by 39%. Compared with Cd-treated group, treatment with organic acids did not change the activities of SOD and CAT (Figure 2).

In the leaves, Cd induced the activities of SOD, POD, and APX by 82, 36, and 210%, respectively, as compared with CK (Figure 3). Citric acid addition reduced the activities of SOD, POD, and APX by 31, 34, and 55%, respectively. Treatment of three organic acids had negligible effect on SOD activity, while they greatly influenced the activities of POD and APX. The addition of tartaric acid significantly reduced the APX activity by 39%, whereas malic acid treatment increased the POD activity by 45%, as compared with the Cd-treated group (Figure 3). CAT activity remained relatively stable except the treatment of tartaric acid showing considerably higher activity of CAT than the CK group.

### Contents of Non-protein Sulfhydryl Compounds

**FIGURE 4:**
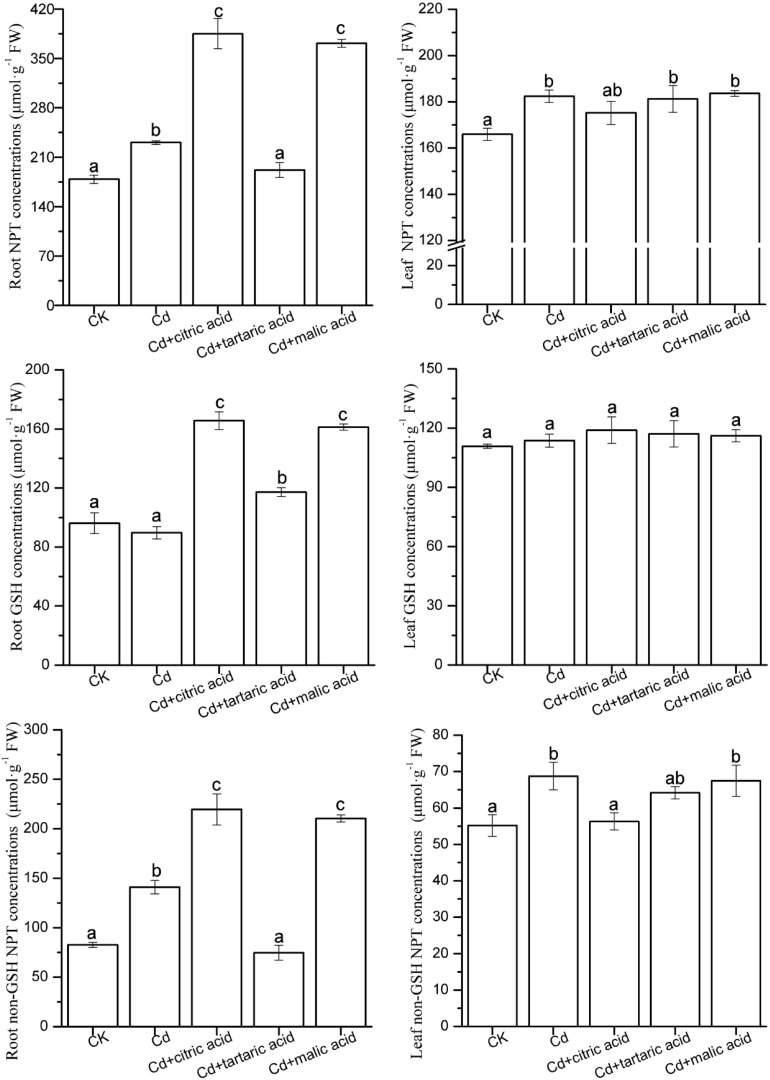
Effects of 100 μM of exogenous organic acid (citric, tartaric, or malic acid) on the concentrations of NPT, GSH, and non-GSH NPT in roots and leaves of *S. variegata* exposed to 50 μM of Cd for 4 weeks. Values are means ± SE (*n* = 3). Different letters indicate significant difference between treatments at *P* < 0.05.

As shown in Figure 4, under Cd stress, the content of total NPT in roots and leaves of *S. variegata* significantly increased by 29 and 10%, respectively, as compared with CK. The non-GSH NPT content in roots and leaves also increased by 71 and 25%, respectively. However, GSH content was not affected by Cd. The addition of citric acid or malic acid markedly increased the content and composition of NPT in roots of *S. variegata* (Figure 4). The contents of NPT, GSH, and non-GSH NPT of roots in citric acid treatment group were 167, 185, and 156% of that in Cd treatment group, respectively, whereas their contents in the malic acid treatment group were 161, 180, and 149% of that in Cd treatment group, respectively. In contrast, tartaric acid treatment showed the opposite tendency (Figure 4). The contents of total NPT and non-GSH NPT in roots significantly decreased as tartaric acid was added, whereas GSH content showed a notable increase compared with the Cd-treated group. Precisely, the contents of NPT, GSH, and non-GSH NPT of roots in the tartaric acid treatment were 83, 131, and 53% of the Cd treatment group, respectively.

In contrast, treatment with each organic acid had little impact on NPT and GSH in leaves of *S. variegata*, except that non-GSH NPT was reduced to 82% of that in the Cd-treated group by citric acid. In general, the contents of NPT and non-GSH NPT in roots of *S. variegata* were higher than that in leaves under each treatment (Figure 4).

### Expression of Cd Accumulation and Tolerance Candidate Genes

**FIGURE 5:**
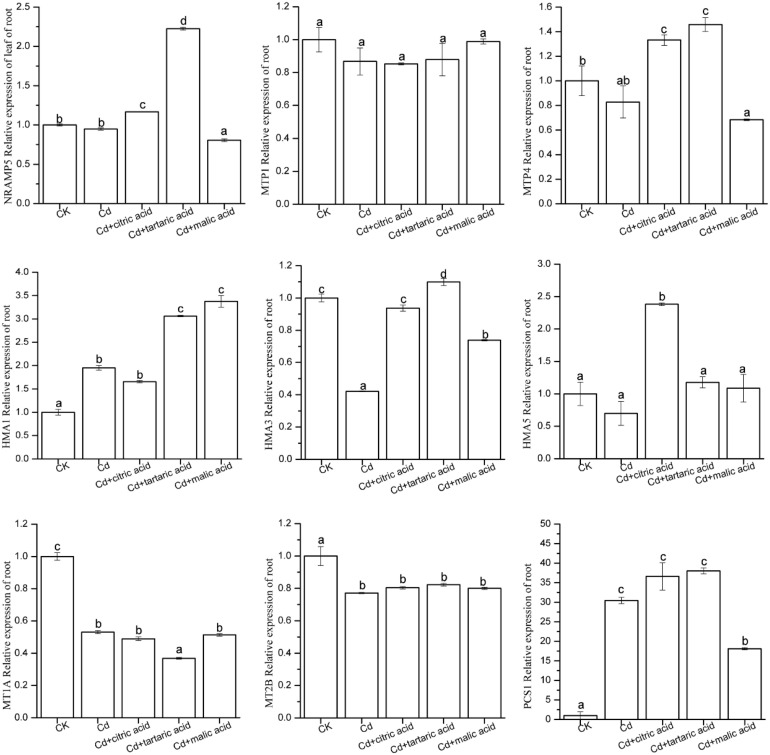
Effect of 100 μM of exogenous organic acid (citric, tartaric, or malic acid) on the expression level of candidate genes related to Cd transportation and detoxification in the roots of *S. variegata* exposed to 50 μM of Cd for 4 weeks. Values are means ± SE (*n* = 3). Different letters indicate significant difference between treatments at *P* < 0.05.

**FIGURE 6:**
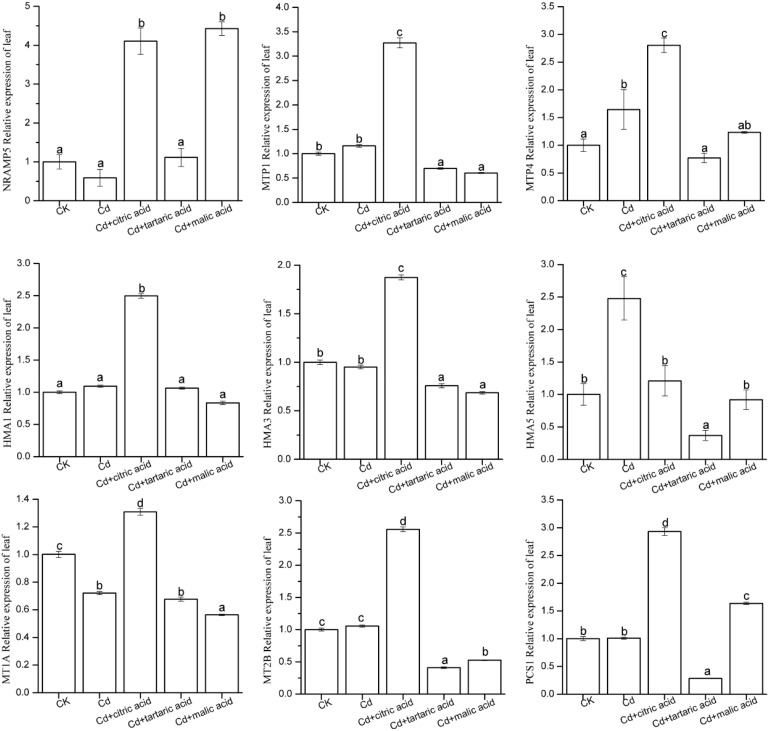
Effect of 100 μM of exogenous organic acid (citric, tartaric, or malic acid) on the expression level of candidate genes related to Cd transportation and detoxification in the leaves of *S. variegata* exposed to 50 μM of Cd for 4 weeks. Values are means ± SE (*n* = 3). Different letters indicate significant difference between treatments at *P* < 0.05.

In order to investigate the effects of organic acids on the expression levels of nine candidate genes involved in metal transport and detoxification in roots and leaves of *S. variegata*, qRT-PCR analysis was performed on these nine genes (Figures 5, 6). In roots, Cd significantly induced the expression level of P-type metal ATPase protein *HMA1* and PC synthase family protein *PCS1*, reaching 195% and 30.45 times of CK, respectively (Figure 5). MT *MT1A*, *MT2B*, and P-type metal ATPase protein *HMA3* were disrupted by Cd, and their expression levels were significantly down-regulated, reaching only 53, 77, and 42% of CK, respectively (Figure 5). The expression level of other candidate genes in Cd treatment group was similar with that in CK. Respective treatment by citric, tartaric, and malic acid had varying effects on the expression levels of nine candidate genes in the root tissues of *S. variegata*. *HMA1* expression was further up-regulated by the addition of tartaric acid or malic acid, reaching 157 and 173% times of that in the Cd treatment group, respectively. *PCS1* was down-regulated under the treatment of malic acid, but still significantly higher than CK. The expression of *HMA3* in roots was greatly up-regulated after the respective treatment with citric acid, tartaric acid, and malic acid, reaching up to 223, 261, and 175% of that in the Cd treatment group, respectively. No significant variation in the expression of *MT1A* and *MT2B* was observed between organic acid treatments and Cd treatment group. The expression levels of *NRAMP5* and *MTP4* increased significantly in the citric acid and tartaric acid treatment groups, whereas *HMA5* was significantly up-regulated only in the citric acid–treated group, and *AtMTP1* did not vary between treatments.

In the leaves, Cd stress significantly increased the expression level of *MTP4* and *HMA5* by 64 and 148%, respectively, as compared with CK (Figure 6). Among the nine candidate genes, only *MT1A* was markedly down-regulated by Cd, holding 72% of CK. The expression level of the other candidate genes in Cd treatment group was similar to that in CK. Respective addition of citric, tartaric, and malic acid greatly influenced the expression of nine candidate genes. In the citric acid treatment group, the expression levels of *NRAMP5*, *MTP1*, *MTP4*, *HMA1*, *HMA3*, *MT1A*, *MT2B*, and *PCS1* were 6.96, 2.82, 1.70, 2.28, 1.97, 1.82, 2.42, and 2.90 times of that in Cd treatment group, respectively. In contrast, the addition of tartaric acid decreased the expression levels of *MTP1*, *MTP4*, *HMA3*, *HMA5*, *MT2B*, and *PCS1* to 60, 47, 80, 15, 39, and 28% of that in Cd treatment group, respectively. The expression levels of *NRAMP5* and *PCS1* were substantially higher in malic acid treatment, reaching 7.51 and 1.62 times of that in Cd treatment group, respectively, whereas the expression levels of *MTP1*, *HMA3*, *HMA5*, *MT1A*, and *MT2B* decreased to 52, 72, 37, 78, and 50% of that in the Cd treatment group, respectively (Figure 6).

## Discussion

Cd is a highly toxic element with no known biological function in plants and can interfere with cellular enzyme system, oxidative stress reaction, and nutrient metabolism within plants (Irfan et al., 2013). Plant resistance to Cd is mainly determined by physiochemical tolerance, metal transport and sequestration, and antioxidant reactions (Jin et al., 2008). Examining the plant physiochemical response to different organic acids addition under Cd stress would improve our understanding of the mechanistic role of organic acids in Cd alleviation and detoxification in plants. The present study showed that the biomass of *S. variegata* exposed to Cd was markedly increased by organic acids, among which the malic acid exerted the most obvious influence. On the other hand, although Cd content in the tissues of *S. variegata* varied with the types of organic acids, the total Cd accumulation in the groups treated with organic acids was still rather higher than that in Cd-treated group. This indicates that application of exogenous organic acids is a promising approach for the phytoremediation of heavy metal contaminated soil. The presence of organic acid in the nutrient solution may alter the speciation of elements and thus plant uptake to Cd and other essential ions can be drastically modified (Najeeb et al., 2011). Therefore, the effect of different organic acids to the absorption of macronutrients, with or without Cd, should be fully examined in the future research. The Cd-induced ROS production is one of the main consequences poisonous to plants. Cd is a non-redox active transition metal that indirectly causes excessive production of ROS by inhibiting the antioxidant defense system or by stimulating membrane-bound NADPH oxidase (Pandey and Singh, 2012; Chmielowska-Bak et al., 2014). Excessive ROS can cause membrane lipid peroxidation, in which the oxygenolysis of lipids containing carbon–carbon double bonds occurs and MDA is produced as the final product. Therefore, increasing MDA content is generally considered to be a hallmark of oxidative damage in plants under metal stress (Najeeb et al., 2011). In this study, Cd treatment resulted in a significant increase in MDA content in roots and leaves of *S. variegata*. Similar results have been reported in *Pisum sativum* (Pandey and Singh, 2012) and *Brassica juncea* (Kaur et al., 2017). Yang et al. (2007) also found that MDA increased in leaf cells of rapeseed plants (*Brassica napus*) under Cd exposure. The content of MDA in the leaves of *S. variegata* was higher than that in the roots, indicating that leaf membrane lipid peroxidation suffered more severely than that of roots under 50 μM Cd. This is consistent with the findings of Li who studied ramie (*Boehmeria nivea*) under Cd stress (Li, 2014). Photosynthesis is the main source of ROS produced in plants. Cd causes degradation of Chl and Car and inhibits their biosynthesis, thereby interfering with the electron transport rate of PSI and PSII and leading to ROS accumulation (Touiserkani and Haddad, 2012). The respective addition of citric acid, tartaric acid, and malic acid alleviated lipid peroxidation in the roots and leaves of *S. variegata*, and the malic acid had the most significant effect.

Antioxidant enzymes play important roles in plant adaptation to abiotic stresses. Enhancement of ROS scavenging, a strategy to cope with Cd stress, can be achieved by the regulation of various antioxidant enzyme activities in plants (Smeets et al., 2008). In this study, Cd stress significantly stimulated the activity of SOD and CAT in roots and SOD, POD, and APX in leaves of *S. variegata*. SOD is an important antioxidant enzyme that provides the first line of defense against superoxide anions by catalyzing the conversion of O_2_^–^ to H_2_O_2_, and O_2_. CAT has high conversion rate and can catalyze the decomposition of H_2_O_2_ into H_2_O and O_2_. Similarly, POD, and APX are also important antioxidant enzymes, both of which promote the decomposition of H_2_O_2_ (El-Beltagi and Mohamed, 2013). The POD activity in the roots of *S. variegata* increased significantly under the respective addition of three organic acids, and leaf POD activity also greatly increased after the malic acid addition. The increase of these enzymatic activities may be attributed to the enhanced expression of relevant antioxidative enzyme genes such as *POD1*, *Cu/Zn-SOD*, and *GPX1* induced by organic acids (Guo et al., 2017). This also indicates that organic acid supply enhances the resistance of *S. variegata* to Cd-caused oxidative stress. However, the tartaric acid treatment significantly reduced APX activity in tissues of *S. variegata*. This may be related to the chelation of Cd by organic acids, which alleviates Cd-induced oxidative stress and ROS, and H_2_O_2_ accumulation would thus greatly decrease (Ali et al., 2015).

The chelation of Cd by sulfhydryl substances (GSH, PC, Cys, etc.) is also one of the dominant detoxification mechanisms in plants. The sulfhydryl group has a strong affinity with Cd, and the formed nontoxic complex can exist in the cytoplasm or be transported to the vacuole, which reduces the toxicity and migration of Cd ions within tissues (Li et al., 2011). The present study showed that increased NPT content in the roots and leaves of *S. variegata* under Cd stress was mainly the increase of non-GSH NPT content, which is consistent with the results of Sun et al. (2005). In this study, the individual addition of citric acid and malic acid significantly increased the non-GSH NPT content in the roots of *S. variegata* under Cd stress but that content in tartaric acid treatment group decreased significantly, which may be associated with the differential effect of organic acids on Cd absorption capacity and chelation ability in this species. GSH, a precursor for PC biosynthesis, participates in reactive oxygen metabolism as a crucial non-enzymatic antioxidant. A rational GSH and PC balance in plants has an important effect on plant tolerance to Cd, and elevated GSH increases the capacity of plants against Cd-imposed oxidant damages (Semane et al., 2007; Hasanuzzaman et al., 2012). In this study, respective addition of three organic acids markedly increased the GSH content in roots of *S. variegata* under Cd stress, indicating that this species could sufficiently produce and supplement the GSH for PC biosynthesis and antioxidant consumption in the existence of organic acids.

In the regard of plant adaptation to heavy metals, woody species and herbaceous species may have similar physiological and molecular mechanisms, which establish the foundation for the absorption, transport, storage, and detoxification of heavy metals (Luo et al., 2016). However, studies of heavy metal transport in plants have shown that woody plants may have more complex mechanisms than herbaceous (Migeon et al., 2010; Yamaguchi et al., 2011). *NRAMPs* maintain the equilibrium state of metals by translocating divalent and trivalent ions in plants. In this study, Cd had little impact on the expression of *NRAMP5* in tissues of *S. variegata*, whereas individual addition of citric acid and tartaric acid significantly increased the expression of *NRAMP5* in the roots, showing a consistent trend of variation in Cd content under organic acid treatment (Table 3). In contrast, the expression level of *NRAMP5* in leaves was significantly improved by the citric acid and malic acid treatment, respectively. Previous studies found that Zn-MTPs such as *AtMTP1*, *PtdMTP1*, *CsMTP1*, *AtMTP3*, and *CsMTP4* were mainly involved in the transportation and sequestration of vacuolar Zn and Cd (Blaudez et al., 2003; Kobae et al., 2004; Migocka et al., 2015). This study showed that the expression of *MTP4* in the roots was considerably boosted by respective addition of citric acid and tartaric acid, which seemed to be associated with Cd absorption enhancement by the root of *S. variegata* (Table 3). *HMAs* play an important role in heavy metal hyperaccumulators and super-resistant plant species (Lee et al., 2007). Under Cd stress, *HMA3* expression level was significantly suppressed and might be damaged. Individual addition of three organic acids increased the expression of *HMA3* in roots and might thus improve plant tolerance. The expression of *HMA1* in *S. variegata* significantly increased under Cd stress, and the tartaric acid and malic acid treatment further promoted the gene expression. Moreover, the addition of citric acid notably boosted the expression of *HMA5* in roots and *HMA1* and *HMA3* in leaves. These results indicate that three exogenous organic acids improved the Cd tolerance of *S. variegata* through the stimulation of *HMA1*, *HMA3*, and *HMA5* expression, especially in the roots of *S. variegata*. The PC peptide is a non–gene-coding product, and the *PCS* of PC synthetase family plays a key role in the synthesis of PC peptides (Picault et al., 2006). In this study, the expression level of *PCS1* in the roots was drastically induced by Cd, which was 30.45 times of that of CK. This suggests that *PCS1* is very likely to be involved in the accumulation of Cd in roots of *S. variegata*. The respective addition of citric acid and tartaric acid further increased the expression of *PCS1*. As a contrast, malic acid addition reduced its expression, but it was still significantly higher than that of CK. In the leaves, the expression level of *PCS1* was also significantly increased in Cd + citric acid and Cd + malic acid treatments. Decreased expression level of *MT1A* and *MT2B* in each treatment group and decreased *PCS1* expression in the Cd + tartaric acid treatment group might be both related to the Cd accumulation in leaves of *S. variegata*.

## Conclusion

This study showed that Cd exposure significantly inhibited plant biomass and provoked the activity of antioxidant enzymes, as well as MDA concentration in the tissues of *S. variegata*. Respective addition of the three organic acids greatly promoted plant resistance to oxidative stress and reduced the Cd-imposed lipid peroxidation, with the effect of malic acid showing greatest. However, citric acid markedly increased the total Cd accumulation of *S. variegata* compared with the other two organic acids. The addition of organic acids also significantly increased GSH content in the root, potentially further improving the antioxidant capacity and PC biosynthesis. Under the stress of Cd, the candidate genes were mainly expressed in the roots of *S. variegata*, and the addition of three organic acids further promoted the expression of candidate genes in *S. variegata*. In conclusion, application of citric, tartaric, or malic acid alleviated Cd-imposed toxicity through the boost of enzymatic and non-enzymatic antioxidants and candidate gene expression, whereas their effects on Cd tolerance and accumulation of *S. variegata* differed.

## Data Availability Statement

The raw data supporting the conclusions of this article will be made available by the authors, without undue reservation.

## Author Contributions

SZ: conceptualization, methodology, visualization, validation, writing–original draft preparation, and writing–reviewing and editing. HC: conceptualization, methodology, investigation, data curation, visualization, writing–original draft preparation, and writing–reviewing and editing. DH: investigation, visualization, data curation, and writing–reviewing and editing. XH: investigation, data curation, and validation. YY: investigation, data curation, and validation. KW: investigation, data curation, and validation. HW: funding acquisition, conceptualization, resources, supervision, project administration, writing–reviewing and editing. All authors: contributed to the article and approved the submitted version.

## Conflict of Interest

The authors declare that the research was conducted in the absence of any commercial or financial relationships that could be construed as a potential conflict of interest.
